# Clinicopathologic characteristics and outcomes of Chinese patients with non‐small‐cell lung cancer and *BRAF* mutation

**DOI:** 10.1002/cam4.1014

**Published:** 2017-01-30

**Authors:** Xi Ding, Zengli Zhang, Tao Jiang, Xuefei Li, Chao Zhao, Bo Su, Caicun Zhou

**Affiliations:** ^1^ Central LaboratoryShanghai Pulmonary HospitalTongji University School of MedicineShanghai200433China; ^2^ Department of RespiratoryThe Second Affiliated Hospital of Soochow UniversitySuzhou215004China; ^3^ Department of Medical OncologyShanghai Pulmonary Hospital & Thoracic Cancer InstituteTongji University School of MedicineShanghai200433China; ^4^ Department of Lung Cancer and ImmunologyShanghai Pulmonary HospitalTongji University School of MedicineShanghai200433China

**Keywords:** BRAF mutation, Chinese, clinicopathologic features, non‐small‐cell lung cancer

## Abstract

*BRAF* mutation is one of the important driver oncogene in non‐small‐cell lung cancer (NSCLC). Data on Chinese patients with *BRAF*‐mutant NSCLC are inadequate. Hence, we conducted this study to investigate the clinicopathologic features and outcomes of Chinese patients with NSCLC and *BRAF* mutations. We identified patients with *BRAF*‐mutant NSCLC between January 2012 and April 2016. Patient characteristics and treatment outcomes were analyzed. In total, 1680 patients were included. Twenty‐eight (1.7%) patients harbored *BRAF* mutations. Compared to patients with non*‐BRAF* mutation, patients with *BRAF* mutations were associated with adenocarcinomas (89.3% vs. 70.6%, *P *=* *0.048) and never smokers (78.6% vs. 56.7%, *P *=* *0.019). There were no significant differences in the age, gender distribution, metastasis, or stage at first diagnosis between two groups. Response rates and progression‐free survival (PFS) were similar between patient with *BRAF* mutations and *EGFR* (5.6 vs. 5.8 months; *P *=* *0.277) or *KRAS* (5.6 vs. 4.7 months; *P *=* *0.741) mutations to first‐line chemotherapy. Compared to patients with non‐V600E mutations, patients with V600E‐mutated tumors had a shorter PFS to first‐line chemotherapy, although this did not reach statistical significance (5.2 vs. 6.4 months; *P *=* *0.561). In multivariate analyses, only ECOG PS remained the independent predictor of overall survival (HR = 0.208; *P *=* *0.004). In conclusion, *BRAF* mutation in Chinese patients with NSCLC was rare. *BRAF* mutation is more likely to be associated with adenocarcinoma and never smokers. *BRAF* mutations are not associated with enhanced chemosensitivity and novel and effective drugs inhibiting the *BRAF* pathway are in urgent need.

## Introduction

Lung cancer is one of the most common malignancies and the leading cause of cancer death worldwide, with 1.6 million new cases and 1.38 million deaths annually 1. The discovery of driver oncogene in a subset of patients with non‐small‐cell lung cancer (NSCLC) has transformed the therapeutic methods to them. Patients with epidermal growth factor receptor (*EGFR*)‐activated mutation or anaplastic lymphoma kinase (*ALK*) fusion obtain significant benefit from targeted therapy with small molecule tyrosine kinase inhibitors (TKIs) 2, 3, 4, 5. With the completion of genomic analysis in lung cancer by The Cancer Genome Atlas (TCGA) Research Network 6, 7, more and more sensitizing molecular alterations have been identified in genes such as *KRAS, ROS1, RET, BRAF*,* HER2, MET exon 14*, and *PIK3CA* that could potentially be targeted in NSCLC 8, 9.

BRAF, one of the serine/threonine protein kinase, belongs to the RAF kinase family in the RAS‐RAF‐MEK‐ERK signaling pathway 10, 11. When activated by mutations, BRAF activates MEK and this leads to the activation of the ERK signaling pathway to promote cell growth, proliferation, and survival 12. The most common mutation in *BRAF* is the valine (V) to glutamic acid (E) substitution at residue 600 (*BRAF* V600E), which results in a mutant BRAF protein that no longer requires dimerization for its activity and is constitutively active and transforming in vitro 13, 14, 15, 16. Somatic mutations in *BRAF* are found in several kinds of cancers, including melanoma, ovarian carcinomas, colorectal cancers, papillary thyroid cancers, and lung cancers. *BRAF* mutations are most commonly seen in melanoma, where *BRAF* V600E is the driver mutation that can be effectively targeted with selective BRAF and/or MEK inhibitors 17, 18, 19, 20. *BRAF* mutations are also observed in 1–3% of NSCLC 21, 22, 23, 24, 25. Studies on lung cancers, in which *BRAF* mutations were observed have generated considerable interest because these mutations may be associated with increased sensitivity to agents directly targeting BRAF or BRAF‐mediated downstream signaling pathways 26, 27. Hence, several previous reports have begun to define the prevalence, distribution, and prognosis of *BRAF* mutations in patients with NSCLC 21, 22, 23, 24, 25, 28. But there are several limitations of the published articles: (1) the enrolled patients were from Europe and America and little study included Chinese patients with NSCLC. As is known, the genetic background between Caucasians and Asians with NSCLC is totally different. (2) limited by relatively small numbers of patients, few study reported the effect of first‐line chemotherapy in NSCLC patients with *BRAF* mutations; (3) they also did not compare the effect of first‐line chemotherapy in *BRAF‐*mutant patients with patients who harbored other activating mutations such as *EGFR* and *KRAS*. We therefore conducted this study with the aim of clarifying the clinicopathologic characteristics and effect of chemotherapy in Chinese patients with *BRAF*‐mutant NSCLC.

Toward this aim, we analyzed arguably the largest cohorts to describe the clinicopathologic characteristics of Chinese patients with *BRAF*‐mutant NSCLC in this study. Meanwhile, we assessed the effect of first‐line chemotherapy in patients with NSCLC and *BRAF* mutations. In addition, we also compared the therapeutic effect of chemotherapy in NSCLC patients who harbored *BRAF* mutations with those who harbored *EGFR* or *KRAS* mutations.

## Materials and Methods

### Patients cohort

Data of patients with pathologically confirmed lung cancer who received *EGFR*,* KRAS*, and *BARF* mutation test at the Thoracic Cancer Institute, Tongji University from January 2012 to April 2016 were retrospectively reviewed. The major clinicopathologic characteristics including sex, age, smoking history, Eastern Cooperative Oncology Group performance status (ECOG PS), lung cancer histology (WHO classification) 29, *EGFR*,* KRAS*, and *BARF* mutation status, metastases and stage were all collected. A never smoker was defined as a person who had smoked <100 cigarettes during his/her lifetime. Age, smoking status, and ECOG PS were documented at first diagnosis. Thoracic Cancer Institute, Tongji University School of Medicine established requirements for clinical information on patient follow‐up under treatment, including response to treatment and survival. Patients were followed from the date of cancer diagnosis until date of death or last available follow‐up. Tumor response was evaluated according to the Response Evaluation Criteria in Solid Tumors version 1.1 (RECIST v1.1), including complete response (CR), partial response (PR), stable disease (SD), or progressive disease (PD). The treatment response was evaluated 1 month after the initiation of therapy and then every 2 months. This study was approved by Shanghai Pulmonary Hospital Ethics Committee and a written informed consent was obtained from each patient to use the clinical data for research before the medical intervention started.

### Molecular analysis

All mutational analyses were conducted at the Thoracic Cancer Institute, Tongji University Medical School, Shanghai. Briefly, DNA from tissue was extracted using the DNeasy Blood and Tissue Kit or the QIAamp DNA FFPE Tissue Kit (both from Qiagen, Hilden, Germany). *EGFR*,* BRAF*, and *KRAS* mutations were tested by amplification refractory mutation system (ARMS) as described in our previous studies (Amoy Diagnostics Co. Ltd., Xiamen, China) 30, 31, 32, 33. *BRAF* mutations were further confirmed by direct sequencing.

### Statistical analysis

The categorical variables were analyzed by chi‐square tests, or Fisher's exact tests when needed. The continuous variable was compared using ANOVA and Tukey's multiple comparison tests. Kaplan–Meier curve and two‐sided log‐rank test were used for univariate survival analyses. Cox proportional hazards model was used for uni‐ and multivariate survival analyses to calculate the hazard ratios (HR) and corresponding 95% confidence intervals (CI). Overall survival (OS) was calculated from the date of lung cancer diagnosis to death from any reasons or was censored at the last follow‐up date. Progression‐free survival (PFS) was defined as the time from the date of first‐line treatment initiation to the date of systemic progression or death and was censored at the date of last tumor assessment. Disease progression was defined in accordance with the RECIST version 1.1. *P* values were considered significant if less than 0.05 (two‐sided). All statistical analyses were performed using the SPSS statistical software, version 22.0 (SPSS Inc., Chicago, IL).

## Results

### Patients’ characteristics

A total of 1680 NSCLC cases who received *EGFR*,* KRAS*, and *BARF* mutation test were identified. All patients were Chinese. The NSCLC patients consisted of 1023 female, 953 never smoker, and 1186 adenocarcinomas. Of the 1680 NSCLC patients, 28 had tumors bearing *BARF* mutation (1.7%), 799 had tumors bearing *EGFR* mutation (47.6%), and 149 had tumors bearing *KRAS* mutation (8.9%). Three mutation genotypes were identified: V600E (*n* = 24), G469A (*n* = 3), G469V (*n* = 1). Four patients with a *BRAF* mutation had a concomitant mutation in *EGFR* (*n* = 3) or *KRAS* mutation (*n* = 1). The baseline and clinical characteristics of all included patients were summarized in Table 1.

**Table 1:** Baseline characteristics of the study population

Variables	All patients	BRAF mutation	EGFR mutation	KRAS mutation	*P* value^a^
Total	1680	28	799	149	
Age at diagnosis					
<65 years	994	15	462	78	0.544
≥44 years	686	13	337	71	
Gender					
Male	657	12	285	109	0.682
Female	1023	16	514	40	
Smoking history					
Never‐smoker	953	22	597	51	0.019
Former/current smoker	727	6	202	98	
ECOG performance status					
0–1	945	20	721	80	0.103
≥2	735	8	78	69	
Pathological classification					
Adenocarcinoma	1186	25	718	122	0.048
Non‐adenocarcinoma	494	3	81	27	
Metastasis at time of diagnosis					
Yes	234	6	163	17	0.248
No	1446	22	636	132	
Stage at diagnosis					
IIIB	956	17	463	75	0.681
IV	724	11	336	74	

a *P* value refers to the comparison of patients with BRAF mutation versus non‐BRAF mutation.

### Clinicopathologic characteristics associated with *BRAF‐*mutant NSCLC

*BRAF* mutations were present in 16 women and 12 men with an average age of 64 years (range, 37–78 years). Twenty‐two patients (78.6%) were never smokers. Histopathologic stage varied and included IIIB (*n* = 17) and IV (*n* = 11). Twenty‐five tumors were adenocarcinomas and three tumors were non‐adenocarcinoma. Six patients had distant metastasis at time of diagnosis (21.4%). Most of them (*n* = 20) had the good performance score (0–1). More details of patients’ *BARF* mutations are listed in Supplemental Table S1. Compared to total patients with non*‐BRAF* mutation, patients with *BRAF*‐mutant tumors were more likely to be never smokers (78.6% vs. 56.7%, *P *=* *0.019). Patients with *BRAF* mutation were associated with adenocarcinoma than those with non*‐BRAF* mutation (89.3% vs. 70.6%, *P *=* *0.048). There were no significant differences in the age, sex distribution, metastasis, or stage at time of diagnosis between patients with *BRAF*‐mutant and *BRAF* wild‐type tumors (Table 1).

### The effect of chemotherapy in patients with NSCLC and *BRAF* mutant

**Figure 1:**
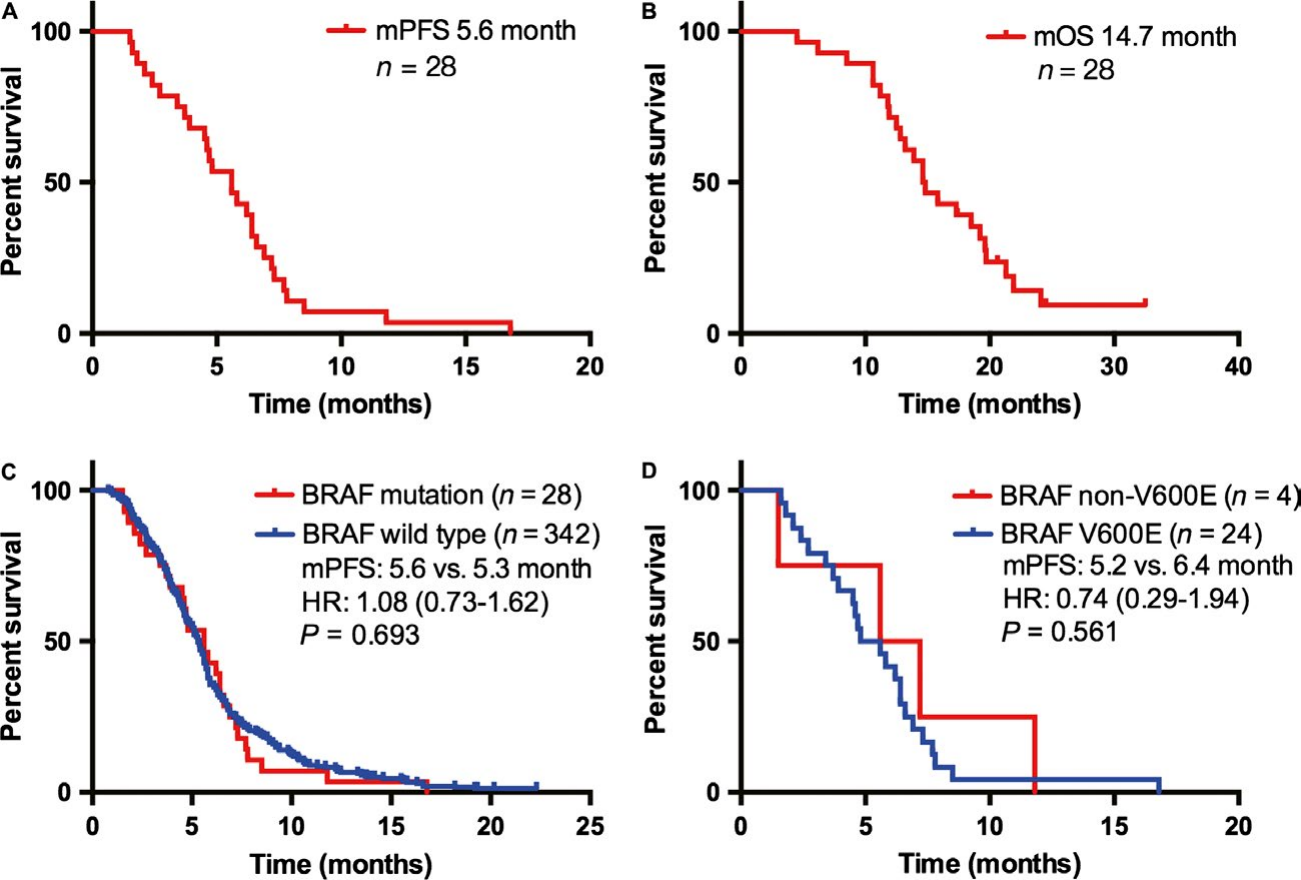
Survival outcomes in Chinese patients with NSCLC and *BRAF* mutation. (A), median progression‐free survival (PFS) of patients who received first‐line platinum‐based combination chemotherapy with NSCLC and *BRAF* mutation; (B), median overall survival of patients with NSCLC and *BRAF* mutation; (C), comparison of median PFS to first‐line chemotherapy between patients with *BRAF* mutations and wild type; (D), comparison of median PFS to first‐line chemotherapy between patients with *BRAF* V600E and non‐V600E mutation. PFS, progression‐free survival.

**Figure 2:**
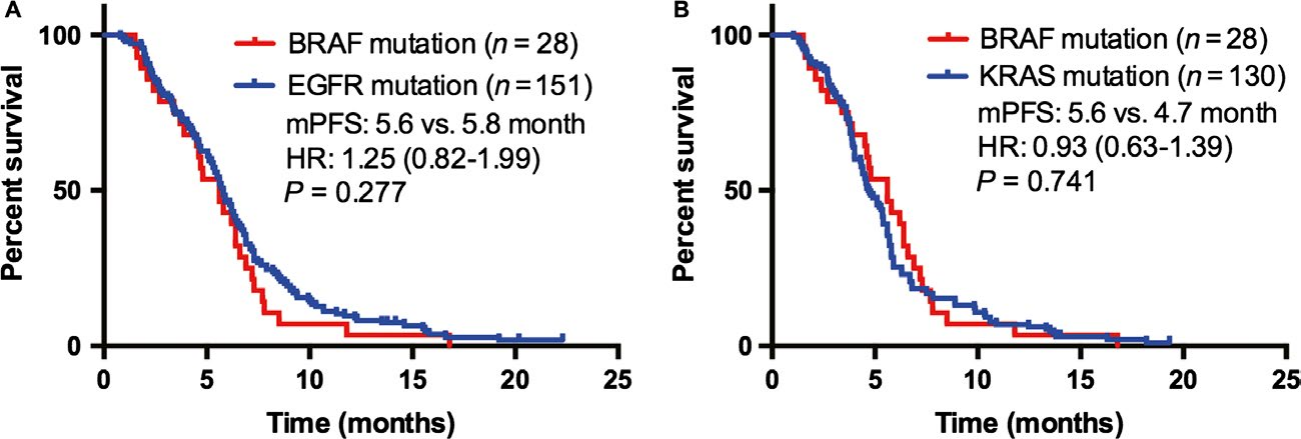
Comparison of median progression‐free survival to first‐line platinum‐based combination chemotherapy in patients with *BRAF* mutations versus *EGFR* (A) or *KRAS* mutations (B).

We determined best response by RECIST 1.1 to first‐line chemotherapy in patients who had adequate scans for radiographic assessments. Within the BRAF cohort, 8 (28.6%) of 28 eligible patients had a PR, 14 (50.0%) had SD, and 6 (21.4%) had PD when treated with platinum‐based chemotherapy. Similar numbers were seen in the EGFR and KRAS cohort: in patients with *EGFR* mutation, 51 (33.8%) of 151 eligible patients had a PR, 70 (46.3%) had stable disease, and 30 (19.9%) had PD; in patients with *KRAS* mutation, 32 (24.6%) of 130 eligible patients had a PR, 61 (46.9%) had stable disease, and 37 (28.5%) had PD (Table 2). There were no significant differences in objective response rate (ORR) and disease control rate (DCR) between patients with *BRAF* mutation and *EGFR* or *KRAS* mutation (Table 2). Figure 1 shows the survival data in these patients. Briefly, median PFS of patients with *BRAF* mutation who received first‐line chemotherapy was 5.6 months (Fig. 1A) compared with 5.3 months for wild‐type patients (*P *=* *0.693; Fig. 1C), and median OS was 14.7 months (Fig. 1B) in patients with *BRAF* mutation. Within *BRAF*‐mutant patients, the median PFS was shorter in patients with V600E mutation compared with non‐V600E mutations, but did not achieve statistical significance (5.2 vs. 6.4 months; HR = 0.74, 95% CI: 0.29–1.94, *P *=* *0.561; Fig. 1D). Compared to patients with *EGFR* mutation, median PFS was similar in patients with *BRAF* mutation who received first‐line chemotherapy (median PFS: 5.6 vs. 5.8 months; HR = 1.25, 95% CI: 0.82–1.99, *P *=* *0.277; Fig. 2A). The median PFS of first‐line chemotherapy was also similar between patients with *BRAF* mutation versus patients with *KRAS* mutation (median PFS: 5.6 vs. 4.7 months; HR = 0.93, 95% CI: 0.63–1.39, *P *=* *0.741; Fig. 2B).

**Table 2:** Response to first‐line chemotherapy in the included patients

	BRAF mutation (*n* = 28)	EGFR mutation (*n* = 151)	KRAS mutation (*n* = 130)	*P* value^a^	*P* value^b^
CR	0	0	0		
PR	8	51	32		
SD	14	70	61		
PD	6	30	37		
ORR	8 (28.6%)	51 (33.8%)	32 (24.6%)	0.591	0.662
DCR	22 (78.6%)	121 (80.1%)	93 (71.5%)	0.850	0.448

CR, complete response; PR, partial response; SD, stable disease; PD, progression disease; ORR, objective response rate; DCR, disease control rate.

a *P* value refers to the comparison of BRAF versus epidermal growth factor receptor mutation.

b *P* value refers to the comparison of BRAF versus KRAS mutation.

### Univariate and multivariate analysis in patients with *BRAF*‐mutant NSCLC

In univariate analysis of the patients with NSCLC and *BRAF* mutation, female patients had marginally significantly longer OS (vs. males; HR = 0.527; 95% CI: 0.193–1.090; *P *=* *0.094). Never smokers had significantly better OS as compared to former/current smoker patients (HR = 0.343; 95% CI: 0.044–0.613; *P *=* *0.011). Patients with ECOG PS 0–1 had significantly longer OS than those with ECOG PS > 1 (HR = 0.279; 95% CI: 0.032–0.367; *P *=* *0.001) (Table 3). No significant difference was found in OS based on age, histology, and co‐occurring driver (e.g., <65 vs. ≥65 lesions, HR = 1.172, *P *=* *0.692; adenocarcinoma vs. non‐adenocarcinoma, HR = 0.390, *P *=* *0.104 and co‐occurring driver vs. no co‐occurring driver, HR = 0.785, *P *=* *0.638) (Table 3). Of note, *BRAF* V600E mutation was not the independent predictor of OS for patients with NSCLC and *BRAF* mutation (HR = 1.737, *P *=* *0.349). In multivariate analyses, only ECOG PS remained the independent predictors of OS. Patients with ECOG PS 0–1 had a significantly lower risk of death than those without (HR = 0.208; 95% CI: 0.071–0.607; *P *=* *0.004) (Table 3).

**Table 3:** Univariate and multivariate analyses of clinical parameters in 28 NSCLC patients with BRAF mutation on overall survival

Factor	Univariate analysis			Multivariate analysis		
	HR (log rank)	95% CI	*P* value	HR (log rank)	95% CI	*P* value
Gender (Female/Male)	0.527	0.193–1.090	0.094	0.590	0.235–1.481	0.261
Age (<65/≥65)	1.172	0.529–2.634	0.692			
Smoking (Never/Smoking)	0.343	0.044–0.613	0.011	0.378	0.117–1.221	0.104
Histology (Adeno/Non‐adeno)	0.390	0.036–1.297	0.104			
PS (0‐1/>1)	0.279	0.032–0.367	0.001	0.208	0.071–0.607	0.004
Co‐occurring driver (Yes/No)	0.785	0.287–2.096	0.638			
BRAF mutation (V600E/non‐V600E)	1.737	0.602–4.468	0.349			

HR, hazard ratio; CI, confidence interval; Adeno, adenocarcinoma; PS, performance score.

## Discussion

To the best of our knowledge, this study was arguably the first large‐scale retrospective study to investigate the clinicopathologic characteristics and outcomes of Chinese patients with NSCLC and *BRAF* mutation. We enrolled 1680 NSCLC patients and 28 of them had *BRAF* mutations. The rate of *BRAF* mutations was 1.7%, which was similar to those reported in Asian populations but lower than those reported in Caucasian populations 22, 25, 28, 34. The relative paucity of *BRAF* mutations in the Chinese patients may be associated with ethnic differences and the high frequency of *EGFR* mutations in Chinese NSCLC patients. Our findings also indicated that NSCLC with *BRAF* mutations are associated with unique clinicopathologic features compared with *BRAF* wild type and other genomic subtypes. In our study, *BRAF* mutations are more likely in never smokers; this is similar to patients with activated *EGFR* and *ALK* alterations, who are also associated with never smokers. In contrast, several previous studies suggested that *BRAF* mutations occurred most often in former/current smokers 25, 28. The possible reason may include that the distribution of *BRAF* mutation types was uneven. In our cohort, 85.7% of patients harbored *BRAF* V600E and only 50–60% of included patients harbored *BRAF* V600E in the previous studies. This was demonstrated by another study, which included 36 lung adenocarcinomas that harbored *BRAF* mutation and showed that *BRAF* V600E was significantly more frequent in never smokers and in female patients, whereas all non‐V600E mutations were detected in smokers 34. Also another two studies based on Chinese population demonstrated that *BRAF* V600E was markedly associated with never smoking and female sex 35, 36. Furthermore, a recent meta‐analysis, which included 10 studies, indicated that there was no significant difference in *BRAF* mutation frequency in former/current smokers versus never smokers (OR = 0.95, 95% CI: = 0.45–2.02), but the difference was significant between former or current smokers and never smokers in patients with *BRAF* V600E (OR = 0.14, 95% CI: = 0.05–0.42) 37. Taken together, we can conclude that *BRAF* V600E mutation is more likely in never smokers, and *BRAF* non‐V600E mutations occur most often in former or current smokers. Specifically, we did not observe an association between gender, age, number of metastases, or stage at time of diagnosis of NSCLC and *BRAF* mutations.

The survival outcomes of patients with *BRAF*‐mutant NSCLC to first‐line chemotherapy closely resembled those with wild‐type tumors. This result was consistent with a previous report that median PFS of NSCLC patients with *BRAF* mutations received platinum‐based combination chemotherapy was similar to patients with *BRAF* wild type (5.2 vs. 6.7 months, *P *=* *0.622) 28. Moreover, the median PFS was similar between patients with *BRAF* mutation and *EGFR*/*KRAS* mutations. These results suggested that *BRAF* mutations are not associated with enhanced chemosensitivity. Compared to patients with non‐V600E mutations, patients with V600E mutations had shorter PFS, although these differences did not reach statistical significance because of low power due to the limited sample sizes. The differences did not seem to be related to imbalances among the subgroups in terms of type of chemotherapy received. Our findings are consistent with three previous reports that showed poor outcomes among patients with *BRAF* V600E mutations compared with *BRAF* wild type 28, 34, 35. Likewise, authors have reported that V600E mutation was frequently related to a more aggressive histotype characterized by micropapillary features 34. Cardarella and colleagues also reported that the median PFS was shorter in patients with V600E mutation compared with non‐V600E mutations, but did not achieve statistical significance (4.1 vs. 8.9 months; *P *=* *0.297) 28. In our study, we did not collect the histological details and we therefore cannot determine the association between micropapillary histology and *BRAF* V600E mutations. To clarify this relationship, future research with large number of cases is warranted.

In our cohort, the co‐occurring driver rate among patients with *BRAF*‐mutant NSCLC was 14.3%. The co‐occurrence of *BRAF* mutations with *EGFR* and *KRAS* mutations has previously been reported in NSCLC, including two patients in the series by Marchetti et al. with concurrent *BRAF* V600E plus *EGFR* mutations and one patient with *BRAF* V600E plus *PIK3CA* mutation and two patients with *BRAF* G464 plus *KRAS* mutations in the series by Cardarella et al. 28, 34. In a study, which enrolled Asian populations, five non‐V600E mutations (four G469A and one G464E/G466R) exhibited concomitant *EGFR* mutations 22. Li et al. reported that five out of eight Chinese patients with lung adenocarcinoma and *BRAF* V600E mutation had concomitant *EGFR* mutations 35. Lung Cancer Mutation Consortium (LCMC) also reported that double‐mutation rate among patients with *BRAF*‐mutant NSCLC was 16% 25. This emphasizes the role of multiplexed genotyping or next generation sequencing in NSCLC genotype because more than one targetable driver mutation may exist within one patient.

To date, two popular second‐generation BRAF inhibitors, dabrafenib and vemurafenib, have shown the promising efficacy in patients with *BRAF* V600E‐mutant NSCLC. In a histology‐independent phase 2 “basket” study, patients with *BRAF* V600 mutation received vemurafenib 38. In the cohort with NSCLC, the ORR was 42% and median PFS was 7.3 months. This is the first time where the efficacy of vemurafenib in NSCLC patients with *BRAF* V600E mutation in the clinical trial has been demonstrated. Then, a phase 2, multicenter, nonrandomized, open‐label study assessed the clinical activity of dabrafenib in patients with NSCLC and *BRAF* V600E mutation 27. The investigator‐assessed ORR was 33% in previously treated patients and 66.7% in previously untreated patients. Furthermore, another recent phase 2, multicenter, nonrandomized, open‐label study investigated the antitumor activity and safety of dabrafenib plus trametinib in patients with *BRAF* V600E‐mutant NSCLC 39. The result showed that combination therapy could achieve a high ORR of 63.2% in previously treated patients. This result indicated that dabrafenib plus trametinib could become a new targeted therapy with robust antitumor activity in these patients. With the publication of these clinical trials, the effectiveness of these BRAF targeted agents would be extensively demonstrated in patients with NSCLC and *BRAF* V600E mutation. In view of the high response rate with dabrafenib plus trametinib in patients with *BRAF* V600E‐mutant NSCLC, future research will investigate the position of dabrafenib plus trametinib as an early treatment option compared with platinum‐based chemotherapy or immunotherapy options.

Our study has several limitations that should be acknowledged. Firstly, despite the initial cohort being large, the number of patients who entered the final analysis was relatively small. Secondly, *BRAF* mutations were detected using ARMS that identified only a limited number of *BRAF* point mutations. We note that other *BRAF* mutations in NSCLC have been identified including mutations in amino acids 421, 439, 459, 466, 471, 595, 597, 604, and 606. Thirdly, although we performed the subgroup analysis of treatment outcomes according to molecular mutations including *BRAF*,* EGFR*, and *KRAS* mutations, compared the *BRAF*‐mutated patients with more specified subgroup. Finally, this study is a retrospective study, which might have induced selection bias. Therefore, the findings in this study need to be validated in prospective trials with large scale.

In summary, this study identified BRAF mutations in 1.7% of Chinese patients with NSCLC. *BRAF* mutation is associated with adenocarcinoma, and *BRAF* V600E mutation is more likely in never smokers. *BRAF* mutations are not associated with enhanced chemosensitivity. This indicates that new and effective drugs targeting the *BRAF* pathway are in urgent need. In addition, NSCLC patients with *BRAF* mutations had the high co‐occurring driver rate. This emphasizes the significance of comprehensive genomic profiling in assessing patients with NSCLC, especially *BRAF*‐mutant NSCLC.

## Conflict of Interest

None declared.

## Supporting information


**Table S1.** Individual characteristics of patients with BRAF‐mutant lung cancer.
