# Supplementary material for: Clinicopathologic characteristics and outcomes of Chinese patients with non‐small‐cell lung cancer and BRAF mutation
Source: Cancer Med. 2017 Jan 30;6(3):555–62. doi: 10.1002/cam4.1014 (PMC5345676; doi:10.1002/cam4.1014)
Supplement: Supplementary file 1 — Table S1. Individual characteristics of patients with BRAF‐mutant lung cancer. [file CAM4-6-555-s001.doc]

| **Supplemental Table S1. Individual Characteristics of Patients With BRAF-Mutant Lung Cancer.** | | | | | | | | | | | | |
| --- | --- | --- | --- | --- | --- | --- | --- | --- | --- | --- | --- | --- |
| Patient | BRAF mutation | Age (year) | Sex | Smoking history | Histological type | ECOG PS | Stage at diagnosis | Co-Occurring Driver | First line treatment | Best response of 1st therapy | PFS of 1st therapy (month) | OS (month) |
| 1 | V600E | 60 | Female | Never | Adenocarcinoma | 0 | IIIB | No | AP | PR | 6.4 | 19.7 |
| 2 | V600E | 62 | Male | Never | Adenocarcinoma | 1 | IIIB | No | AP | SD | 4.5 | 17.4+ |
| 3 | V600E | 48 | Male | Current | Adenocarcinoma | 2 | IIIB | No | AC | PD | 2.1 | 11.9 |
| 4 | V600E | 62 | Female | Never | Adenocarcinoma | 1 | IIIB | No | AP | PR | 7.3 | 19.6 |
| 5 | V600E | 65 | Female | Never | Adenocarcinoma | 0 | IV | EGFR L858R | Gefitinib | SD | 7.7 | 24.1 |
| 6 | V600E | 69 | Male | Former | Adenocarcinoma | 1 | IIIB | No | AC | SD | 4.8 | 13.9 |
| 7 | V600E | 67 | Male | Never | Adenocarcinoma | 1 | IV | No | TC | PR | 6.6 | 18.5 |
| 8 | V600E | 47 | Male | Never | Squamous cell carcinoma | 2 | IV | No | GC | PR | 6.4 | 14.6 |
| 9 | V600E | 58 | Female | Never | Adenocarcinoma | 1 | IIIB | EGFR 19DEL | Gefitinib | SD | 3.9 | 10.6 |
| 10 | V600E | 57 | Female | Never | Adenocarcinoma | 1 | IV | No | AP | SD | 3.7 | 11.2 |
| 11 | V600E | 64 | Male | Never | Adenocarcinoma | 1 | IIIB | No | AP | SD | 5.8 | 17.3 |
| 12 | V600E | 61 | Male | Never | Adenocarcinoma | 1 | IV | KRAS mutation | AC | PD | 1.8 | 6.2 |
| 13 | V600E | 71 | Male | Former | Squamous cell carcinoma | 2 | IIIB | No | GC | PD | 1.6 | 8.5 |
| 14 | V600E | 64 | Female | Never | Adenocarcinoma | 1 | IIIB | EGFR 19DEL | Gefitinib | SD | 3.4 | 11.8 |
| 15 | V600E | 73 | Female | Never | Adenocarcinoma | 1 | IV | No | AP | PR | 8.5 | 19.2 |
| 16 | V600E | 74 | Male | Former | Adenocarcinoma | 1 | IIIB | No | AP | SD | 6.2 | 14.6 |
| 17 | V600E | 60 | Male | Never | Adenocarcinoma | 2 | IV | No | AC | PD | 2.7 | 10.6 |
| 18 | V600E | 68 | Male | Former | Adenocarcinoma | 1 | IIIB | No | AC | SD | 4.6 | 12.8 |
| 19 | V600E | 37 | Female | Never | Adenocarcinoma | 0 | IIIB | EGFR 19DEL | Gefitinib | PR | 16.8 | 32.5+ |
| 20 | V600E | 65 | Male | Never | Adenocarcinoma | 1 | IV | No | TC | SD | 6.9 | 21.3 |
| 21 | V600E | 48 | Female | Current | Adenocarcinoma | 2 | IIIB | No | AP | PD | 2.4 | 12.5 |
| 22 | V600E | 38 | Female | Never | Adenocarcinoma | 1 | IV | No | AC | PR | 7.8 | 20.6+ |
| 23 | V600E | 71 | Female | Never | Squamous cell carcinoma | 2 | IIIB | No | GP | SD | 4.7 | 13.2 |
| 24 | V600E | 70 | Female | Never | Adenocarcinoma | 1 | IIIB | No | AC | SD | 5.6 | 15.8 |
| 25 | G496A | 78 | Female | Never | Adenocarcinoma | 2 | IV | No | AP | PD | 1.5 | 4.5 |
| 26 | G496A | 62 | Female | Never | Adenocarcinoma | 2 | IV | No | AC | SD | 5.6 | 14.8 |
| 27 | G496A | 66 | Female | Never | Adenocarcinoma | 1 | IIIB | No | AP | PR | 11.8 | 24.5+ |
| 28 | G496V | 68 | Female | Never | Adenocarcinoma | 1 | IIIB | No | TC | SD | 7.2 | 21.9 |
| ECOG PS,Eastern Cooperative Oncology Group Performance status; PFS, progression-free survival; OS, overall survival; A, pemetrexed; P, cisplatin; C, carboplatin; G, gemcitabine; T, paclitaxel; PR, partial response; SD, stable disease; PD, progression disease. | | | | | | | | | | | | |
